# Predicting Adherence to Computer-Based Cognitive Training Programs Among Older Adults Using Source-Free Domain Adaptation: Algorithm Development and Validation

**DOI:** 10.2196/79123

**Published:** 2026-03-18

**Authors:** Ronast Subedi, Shayok Chakraborty, Zhe He, Yuanying Pang, Shenghao Zhang, Mia Liza Lustria, Neil Charness, Walter Boot

**Affiliations:** 1 Department of Computer Science Florida State University Tallahassee, FL United States; 2 School of Information Florida State University Tallahassee, FL United States; 3 College of Medicine Florida State University Tallahassee, FL United States; 4 Division of Geriatrics and Palliative Medicine Weill Cornell Medicine New York, NY United States

**Keywords:** source-free domain adaptation, adherence prediction, cognitive training, data privacy, artificial intelligence, AI

## Abstract

**Background:**

Cognitive decline in the aging population presents an unprecedented challenge worldwide. Recent research has shown the potential of cognitive training programs to mitigate cognitive decline. However, these interventions require sustained adherence to be effective, which can be challenging.

**Objective:**

In this study, we aim to enhance the accuracy of predicting adherence patterns in cognitive training programs for older adults, with the goal of developing personalized support systems that promote adherence and improve cognitive outcomes.

**Methods:**

A major challenge in developing deep neural networks for predicting adherence patterns is the limited availability of individual participants’ training data. Although domain adaptation techniques can address this issue by leveraging training data from other clinical studies, our research considers a more practical scenario where the use of such data from other studies is restricted due to privacy and confidentiality concerns. Therefore, we used source-free domain adaptation (SFDA), which uses models trained on other cognitive studies without requiring access to the corresponding datasets. To the best of our knowledge, this is the first effort to use SFDA to predict older adults’ daily adherence to cognitive training programs.

**Results:**

Using data from 3 previously conducted cognitive training intervention studies, our results demonstrated the efficacy of deep learning models combined with SFDA to accurately predict adherence lapses while addressing data privacy concerns.

**Conclusions:**

Our findings indicate that deep learning and SFDA techniques can be useful in the development of adherence support systems for computerized cognitive training, aimed at improving the health and well-being of older adults.

## Introduction

### Background

Cognitive decline presents significant challenges for older people, impacting their well-being, decision-making, and overall quality of life. Although some cognitive decline is normal with age, severe cognitive impairments such as dementia and Alzheimer disease present greater risks [1]. Dementia currently ranks as the seventh leading cause of global deaths, and its prevalence could triple by 2050 [2]. The number of older adults is increasing substantially, particularly among those aged 85 years and older, who represent the fastest-growing age category among older adults [3]. With the US population aged 65 years and older expected to nearly double in the next 40 years [4], addressing age-related cognitive changes is essential for promoting independence, improving quality of life, and reducing caregiving burdens on families and health care systems.

Cognitive training interventions have emerged as a promising approach to address cognitive decline. These interventions often involve technology-based training programs that target specific cognitive functions such as memory, attention, and problem-solving [5-8]. Recent studies have underscored the potential of digital cognitive training programs in improving cognitive performance and daily activities in older adults [9-11]. Lövdén et al [12] assert the following in their theoretical account of the necessary conditions for inducing cognitive plasticity: (1) mismatches between functional supply and experienced environmental demands drive manifestations of plasticity and (2) mismatches need to be maintained for extended periods to induce plasticity. Keeping people engaged and adherent for extended periods while performing tasks that force them to operate slightly above their cognitive limits is a major challenge. A necessary first step in promoting adherence is the ability to predict lapses and to find effective ways to intervene “just-in-time” to maintain engagement.

Recently, Li et al [13] conducted a meta-analysis that included 55 randomized controlled trials with 4455 participants (spanning normal cognition, mild cognitive impairment, and neurodegenerative dementia) to assess the effects of cognitive training in older adults. The study demonstrated that participants who maintained an adherence rate of 80% or higher experienced significant cognitive benefits. This systematic review further highlights the critical role of sustained adherence in augmenting the efficacy of cognitive training and underscores the importance of predicting adherence lapses. Studies also indicate that adherence to cognitive training is influenced by factors such as mental capacity, physical function, and program design [14-19]. Hence, understanding adherence to these interventions and developing strategies to enhance them are essential for improving the effectiveness and optimizing outcomes of computer-based cognitive training programs [20,21].

To this end, in our recent research, we have applied advanced machine learning (ML) and deep learning (DL) techniques to predict adherence lapses based on past behaviors among older adults participating in cognitive training programs [22,23]. Deep neural networks, together with techniques such as data augmentation, adaptive window size estimation, and temporal data clustering, have shown tremendous promise in predicting adherence patterns [24]. To capture the individual differences across participants, we trained a separate prediction model for each participant rather than a single model for all participants. However, the limited availability of data for each participant (only 30 days) made it difficult to train a reliable prediction model for each participant. In our recent work [25], we leveraged the potential of domain adaptation (DA) to address this challenge by using training data from participants in a similar, previously conducted clinical trial. Although the results were promising, this method required access to data from other participants in a different trial, which can raise privacy concerns, as health care data can contain personal or confidential information.

In this study, we used data from 3 technology-based cognitive intervention studies to predict adherence patterns among participants while also addressing data privacy issues. Although these datasets are deidentified, previous publications have shown that deidentification of health data, commonly used to protect privacy, is ineffective because modern techniques can easily reidentify individuals by linking deidentified datasets with external information [26,27]. Similarly, ML techniques can successfully reidentify individuals from incomplete datasets with high likelihood [28]. Despite these limitations, deidentified data are widely shared and sold without patient consent, highlighting the need for stronger protection and privacy practices in health care research.

In this work, we used state-of-the-art source-free domain adaptation (SFDA) techniques to address the challenge of limited training data for each participant and the privacy concerns surrounding clinical data. Our framework requires access only to a *deep neural network model trained on data from a different clinical study, and not the confidential data themselves*, thereby addressing data privacy concerns. Our extensive empirical analyses demonstrated that SFDA can efficiently leverage knowledge from other clinical studies to develop participant-specific deep neural network models to predict adherence patterns, while addressing data privacy issues at the same time.

Overall, our research highlights the potential of deep neural networks and SFDA techniques in predicting adherence lapses and developing personalized support systems for cognitive training programs. SFDA not only improves the prediction accuracy but also preserves the privacy of clinical datasets, which is crucial for confidentiality. By leveraging these technologies, we can enhance engagement and optimize the benefits of cognitive interventions, ultimately improving the quality of life for older people with cognitive impairments. To the best of our knowledge, this study represents the first effort to apply SFDA techniques to predict daily adherence among older adults participating in cognitive training programs.

### Objectives

This research sought to address the following objectives:

1. Determine the potential of convolutional neural networks (CNNs) to predict adherence to cognitive training programs based on past behavior
2. Explore the use of SFDA techniques to address training data scarcity and maintain data privacy while predicting adherence patterns
3. Investigate how artificial intelligence (AI)–driven approaches can be implemented to predict engagement in cognitive training programs

Overall, by identifying adherence patterns, these approaches can inform the development of more effective interventions, benefiting individuals and society by improving conditions associated with aging.

## Methods

### Research Design

For this study, we investigated older adults’ engagement with Mind Frontiers (Aptima Inc), an Android-based cognitive training game platform. The platform features a Wild West–themed interface with 7 interactive minigames designed to enhance key cognitive functions, including memory, attention, spatial navigation, multitasking, and logical reasoning. Cognitive training was conducted on Lenovo 10 tablets, with all participants receiving instructions on device operation and gameplay. The levels reached in the games ranged from 1 to 58, and the cognitive training program included 5 possible outcomes: *defeat*, *stalemate*, *victory*, *abort*, and *not yet finished*. After completing each game, participants received feedback, and the game’s difficulty was adjusted based on their previous performance. This structured approach ensured consistent engagement and systematic data collection for assessing cognitive improvement over time.

### Datasets

In this study, we used 3 distinct datasets, each derived from separate clinical research trials. The study 1 dataset [29] consisted of a 2-phase protocol. In the first phase of 12 weeks, participants were asked to follow a structured schedule, engaging in cognitive training for 45 minutes per day, 5 days per week. In contrast, the subsequent 6-week phase was unstructured, allowing participants to engage at their discretion. In our study, we analyzed data only from the structured phase, as adherence metrics rely on the prescribed game-playing schedule. The dataset is derived from 118 participants with a mean age of 72.6 (SD 5.5) years, including 78 (66.2%) female participants and 40 (33.8%) male participants. The dataset included multiple cognitive assessments, such as technical proficiency, self-efficacy, subjective cognition, perceived benefits, and objective cognitive measures such as processing speed and memory.

The study 2 dataset [30] came from a similar but independent cognitive training study that involved 120 adults aged 64 years and older from Leon County, Florida, and surrounding areas. Of these, 116 (96.7%) participants completed the structured phase, which included baseline and postintervention cognitive measures. The average age of participants was 70 (SD 4.42) years, with a range of 64 to 84 years; 77 (64.2%) participants were female, while 43 (35.8%) participants were male. Although participants were expected to complete both phases, the second phase did not require participants to engage in gaming interventions for specific time intervals.

The study 3 dataset also came from an independent cognitive training trial consisting of 92 participants. Among these, 90 (97.8%) participants provided complete demographic information, with ages ranging from 62 to 84 years and an average age of approximately 71 (SD 4.97) years. Of the participants, 67 (74.4%) were female and 20 (22.2%) were male, while 2 (2.2%) participants preferred not to disclose their sex, and 1 (1.1%) participant indicated that none of the listed sex options described them.

### DL for Adherence Prediction

In this study, we used deep CNNs [31], which were initially designed for image data but are also widely used for time-series classification and forecasting tasks [32,33]. CNNs consist of convolutional layers comprising filters that slide over the input data to extract local patterns and features. For 1D time-series data, 1D convolution is applied, in which the kernel slides unidirectionally from the start of the time series to its end. For an input vector *f* of length *n* and a kernel *g* of length *m*, the convolution (*f* × *g*) of *f* and *g* is defined as follows:




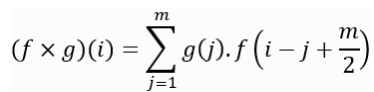




**Figure 1:**
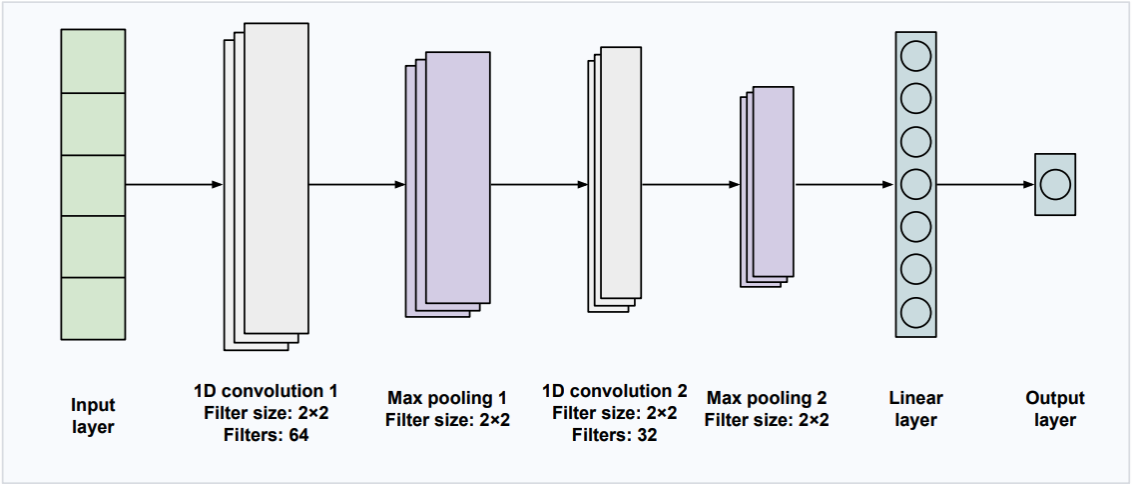
Network architecture used in our experiments. It consists of 2 convolutional blocks, each comprising a convolutional layer followed by a max pooling layer. These blocks are succeeded by a linear layer and an output layer.

In this study, we used a DL architecture consisting of 2 convolutional blocks, each comprising a convolutional layer followed by a maximum pooling layer. The pooling layer down-samples the feature maps by selecting the maximum value within a predefined window, thus effectively retaining salient features. These blocks are succeeded by a linear layer and an output layer, as depicted in Figure 1.

### SFDA for Adherence Prediction

A key challenge in our study was the limited availability of labeled training data for each participant (only 30 days of training data). DA is a well-researched technique in the ML literature for addressing the challenge of training models with limited labeled data. DA algorithms use labeled data from a source domain to enhance the performance of DL models in a target domain of interest with limited labeled data, under the constraint that the data from the 2 domains are derived from different probability distributions [34,35]. Although DA is predominantly used in computer vision, recent studies have demonstrated its applicability in adapting temporal data patterns [36,37].

In our recent research [25], we studied the performance of adversarial DA [38] to enhance the accuracy of personalized adherence prediction models by using training data from participants in a different, previously conducted clinical study. For a given target participant, all participants from the other study, with similar play patterns (determined through a clustering algorithm), were considered source participants. Each participant has a unique play pattern; that is, the data for each participant are derived from a different probability distribution. Adversarial DA was used to address this disparity between the source and target domains, so that the data from the source participants can also be used to train a personalized model for the target participant. Our results demonstrated that DA consistently produced more accurate personalized adherence prediction models for each participant, compared with the case when only the data from the target participant was used to train the model for this participant, without using the data from the other clinical study.

Although these results were promising, the DA algorithm required access to the source participants’ data from the other clinical trial. This can pose privacy concerns as clinical data typically contain personal or confidential information. In this paper, we considered a more practical scenario, assuming we would only have access to a *deep neural network model trained on the source participants’ data, rather than the raw data*. We attempted to leverage the source domain knowledge encoded in the trained source model to train an accurate adherence prediction model for a given target participant. This category of DA is referred to as SFDA [39-45].

Although much SFDA research focuses on computer vision, recent studies have extended SFDA approaches to medical and time-series domains. For instance, Ragab et al [39] introduced a temporal imputation strategy tailored for time-series SFDA, demonstrating improved performance on physiological datasets such as human activity recognition and sleep stage classification. Similarly, studies by Li et al [42] and Wu et al [43] applied SFDA techniques for medical image segmentation, including applications to fundus imaging. Moreover, Yang et al [44] explored the use of SFDA with Fourier-style feature mining for tasks such as polyp and prostate segmentation.

**Figure 2:**
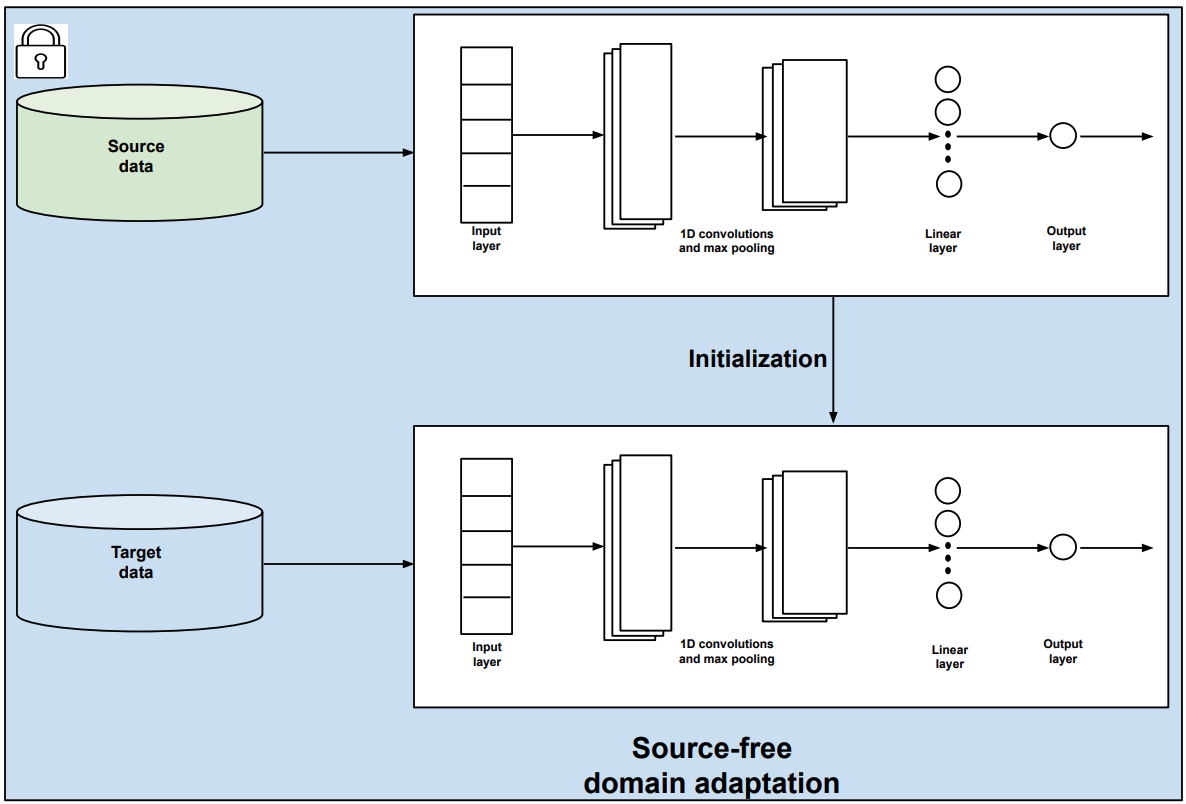
Illustration of source-free domain adaptation (SFDA). We only have access to a deep neural network model trained on the source participants’ data, rather than the raw data. SFDA is effective in addressing data privacy concerns, as only a model trained on source-domain data is used for knowledge transfer to the target-domain participants, rather than the raw source-domain data.

SFDA is effective in addressing data privacy concerns, as only a model trained on source domain data is used for knowledge transfer, rather than the raw source domain data. This is depicted in Figure 2. Our framework thus offers a safer mechanism for clinical trials to inform one another. In this work, we demonstrate the utility of our method using the 3 datasets mentioned as test cases. Our framework will be especially useful for datasets that contain even more sensitive information, given the ease of reidentifying the deidentified data as discussed previously.

**Figure 3:**
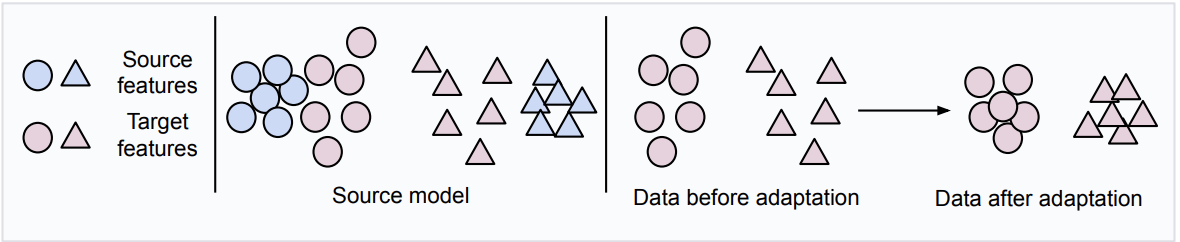
Illustration of local structure clustering (LSC) for SFDA (as described in the studies by Yang et al [40,45]). Some target features from the source model will deviate from dense source feature regions due to domain shift. LSC aims to cluster target features based on their semantically close neighbors.

We used in our study the SFDA method proposed by Yang et al [40]. This method performs adaptation to the target domain without access to source domain data by using local structure clustering (LSC). The main rationale of this method is that although the features of the target domain can be shifted with respect to the features of the source domain, the classes will still form clusters in the feature space. Thus, LSC attempts to move clusters of data points toward their most likely class prediction. Before adaptation, some target features given by the source model are expected to deviate from the corresponding dense source feature region due to domain shift. These samples will most likely be incorrectly predicted by the classifier. However, we assumed that the target features from the same class would be clustered together. Therefore, the nearest neighbors of target features would have a high probability of having the same class labels. To exploit this fact, LSC encourages samples that are close in the feature space to have similar predictions to those of their nearest neighbors. Consequently, clusters of points that are close in feature space will move jointly toward a common class. Thus, this process can correctly classify target features that would otherwise have been wrongly classified. Additional information about this method can be found in the study by Yang et al [40]. Figure 3 presents a visual illustration of the LSC method. To further validate the effectiveness of SFDA, we also conducted an additional experiment using the SFDA method proposed by Yang et al [45] in our setting.

### Experimental Setup

For all the experiments, we used the network architecture illustrated in Figure 1. The network was trained for up to 130 epochs, with early stopping applied when the validation loss ceased to decrease. We used the Adam optimizer with an initial learning rate of 1×10^–3^, which is reduced by a factor of 0.5 every 20 epochs. All models are trained on an Ubuntu 20.04 system equipped with a 20 GB NVIDIA RTX A4500 graphics processing unit. The training of a single model takes approximately 45 minutes. We set aside 15% of the training data for validation and monitored the validation loss to ensure proper model convergence.

In our experiments, we divided each participant’s data into 2 segments for training and testing. The first 30 days of data were considered for training, and the subsequent 30 days of data were used for testing. This division aligns with psychological research indicating that approximately 30 days is the median time required for habit formation to plateau [46].

The input to the CNN consisted of multivariate time-series data, including (1) play duration, (2) session count, (3) maximum level achieved, and (4) number of tasks completed. We established a threshold of 10 minutes of playing time per day to classify participants as adherent (*playing time ≥10 minutes*) or nonadherent, consistent with our previous work [22,24]. Our objective was to train deep neural networks to predict adherence on the (*N* + 1)th day based on data from the previous *N* days. We set the window size, N, to 7 in this study. A personalized model was trained for all participants to capture their unique playing patterns. We used accuracy, precision, recall, and *F*_1_-scores as the metrics for evaluation. Precision, recall, and *F*_1_-scores were used to assess the model’s performance on positive predictions, whereas accuracy evaluated overall performance. The reported final scores represented the average performances across all participants. We conducted 2 different sets of experiments and performed a 1-tailed *t* test to assess the statistical significance of the obtained results. The sets of experiments were as follows: first, no source; in this setting, an individual DL model was trained for each target participant using only the training data of that participant. No information from any other dataset was used for model training. Second, SFDA; here, the SFDA method [40] was used to train personalized DL models for each target participant by transferring relevant knowledge from a source model trained on a source dataset.

For instance, when study 1 served as the source dataset, we first trained a deep model on all the participants’ data from study 1. If the target participant belonged to study 2, the deep model was fine-tuned using data from all other participants in study 2, excluding the target participant. Subsequently, only the trained model, without direct access to the original study 1 dataset, was used to transfer relevant knowledge for developing a personalized adherence prediction model for the target participant.

### Ethical Considerations

This study was approved by the Florida State University Institutional Review Board (study ID 2017.20622 and STUDY00000005). Informed consent was obtained from all participants, and participants were given the opportunity to opt out at any time. This consent process included institutional review board–approved language stating that the collected data could be made available for secondary analysis by other research teams and that any shared data would not include personal identifiers. Data were labeled with participant IDs, meaning that the data were deidentified in this study. Participants were compensated a total of US $200 (study 1) or US $75 (study 2) for their participation, with the difference driven by the shorter commitment involved in study 2.

## Results

The results of our study are presented in Tables 1-3 for the study 1-3 datasets, respectively (using the SFDA method proposed in the study by Yang et al [40]). Each table reports the values of the evaluation metrics on the test sets of the respective datasets. The values reported are the average scores across all participants. The tables show that performance across all the metrics was lowest when no DA technique was applied.

**Table 1:** Average scores of source-free domain adaptation (SFDA) models on the study 1 dataset^a^.

Experiment type	Accuracy (%), mean (SD)	Precision (%), mean (SD)	Recall (%), mean (SD)	*F*_1_-score (%), mean (SD)
No source	63 (0.24)	58 (0.23)	57 (0.24)	54 (0.25)
SFDA with the study 2 dataset as the source	69 (0.20)	63 (0.20)	61 (0.19)	60 (0.21)
SFDA with the study 3 dataset as the source	65 (0.23)	58 (0.23)	56 (0.22)	56 (0.20)

^a^The scores are calculated by averaging the respective scores across all the participants.

**Table 2:** Average scores of source-free domain adaptation (SFDA) models on the study 2 dataset^a^.

Experiment type	Accuracy (%), mean (SD)	Precision (%), mean (SD)	Recall (%), mean (SD)	*F*_1_-score (%), mean (SD)
No source	57 (0.22)	54 (0.19)	52 (0.20)	49 (0.20)
SFDA with the study 1 dataset as the source	68 (0.20)	57 (0.21)	58 (0.19)	56 (0.21)
SFDA with the study 3 dataset as the source	64 (0.21)	55 (0.23)	57 (0.20)	54 (0.20)

^a^The scores are calculated by averaging the respective scores across all the participants.

**Table 3:** Average scores of source-free domain adaptation (SFDA) on the study 3 dataset^a^.

Experiment type	Accuracy (%), mean (SD)	Precision (%), mean (SD)	Recall (%), mean (SD)	*F*_1_-score (%), mean (SD)
No source	56 (0.20)	53 (0.19)	51 (0.22)	50 (0.23)
SFDA with the study 2 dataset as the source	62 (0.20)	61 (0.22)	58 (0.18)	56 (0.21)
SFDA with the study 1 dataset as the source	65 (0.21)	62 (0.25)	59 (0.19)	58 (0.23)

^a^The scores are calculated by averaging the respective scores across all the participants.

For instance, in the study 2 dataset (Table 2), the average accuracy was 57% when only the data for a given target participant was used to train a model for that participant, without access to any other source dataset. This shows that using only the limited participant-specific training data may result in prediction models with poor generalization capability. Using information from the other datasets through SFDA consistently produced better performance in terms of all the evaluation metrics.

For the study 2 dataset, using the study 1 dataset as the source yielded an accuracy of 68% (11% improvement), whereas using the study 3 dataset as the source produced an accuracy of 64% (7% improvement). The corresponding improvements on the *F*_1_-score were 7% and 5%, respectively. A similar pattern was observed for the other 2 datasets.

For the study 1 dataset, using the other 2 datasets as the source datasets resulted in an improvement of 6% and 2% in accuracy and 6% and 2% in the *F*_1_-scores, respectively. For the study 3 dataset, the improvements in accuracy and *F*_1_-score were 6% and 9%, and 6% and 8%, respectively, when using the other 2 datasets as a source, compared with the “No source” setting.

Similarly, using the SFDA technique proposed in the study by Yang et al [45], the improvements in accuracy and *F*_1_-score were 3% and 4%, and 6% and 7%, respectively, compared with the “No source” setting for the study 3 dataset, as depicted in Table 4. The greater performance gains observed when using the study 1 or study 2 datasets as the source dataset, compared with the study 3 dataset, are attributed to the larger sample size of the study 1 (118 participants) and study 2 (116 participants) datasets, compared with the study 3 dataset (92 participants).

**Table 4:** Average scores of source-free domain adaptation (SFDA) on the study 3 dataset (calculated by the method proposed in the study by Yang et al [45])^a^.

Experiment type	Accuracy (%), mean (SD)	Precision (%), mean (SD)	Recall (%), mean (SD)	*F*_1_-score (%), mean (SD)
No source	56 (0.20)	53 (0.19)	51 (0.22)	50 (0.23)
SFDA with the study 1 dataset as the source	59 (0.22)	56 (0.23)	55 (0.19)	56 (0.20)
SFDA with the study 2 dataset as the source	60 (0.23)	58 (0.24)	57 (0.20)	57 (0.25)

^a^The scores are calculated by averaging the respective scores across all the participants.

These results unanimously corroborate the potential of SFDA algorithms to not only develop more accurate personalized DL models for predicting adherence to cognitive training programs among older adults but also help address privacy and confidentiality concerns that are inherent in clinical datasets.

Additionally, we conducted statistical tests of significance using *t* tests (Table 5) and the Wilcoxon signed-rank test (Table 6) to assess whether the improvement in performance achieved by our SFDA method is statistically significant when compared with the *“no source”* method. We computed the *P* values on accuracy scores for all the experiments detailed in Tables 1-3. Tables 5 and 6 show that all *P* values were ≤.05 for all tests, signifying that the performance improvement achieved by the SFDA method over the *“no source”* method is statistically significant. These results further support the effectiveness of SFDA in improving the performance of DL models for predicting adherence to cognitive training programs among older adults while maintaining data privacy and confidentiality.

**Table 5:** *P* values for the accuracy scores obtained by performing a t test comparing the results of the source-free domain adaptation (SFDA) method with the “no source” method.

Datasets		*P* value
**Study 1 dataset**		
	Source: study 2 dataset	.006
	Source: study 3 dataset	.05
**Study 2 dataset**		
	Source: study 1 dataset	.03
	Source: study 3 dataset	.03
**Study 3 dataset**		
	Source: study 1 dataset	.04
	Source: study 2 dataset	.009

**Table 6:** *P* values for the accuracy scores obtained by performing the Wilcoxon signed-rank test comparing the results of the source-free domain adaptation (SFDA) method with the “no source” method.

Dataset			*P* value
**Study 1 dataset**			
	Source: study 2 dataset	.005	
	Source: study 3 dataset	.03	
**Study 2 dataset**			
	Source: study 1 dataset	.02	
	Source: study 3 dataset	.01	
**Study 3 dataset**			
	Source: study 1 dataset	.02	
	Source: study 2 dataset	.008	

## Discussion

### Principal Results

The findings of this study have several significant implications for adherence prediction in cognitive training programs for older adults. Our results demonstrate that CNNs can analyze behavioral patterns and predict adherence trends in cognitive training interventions. CNNs learn meaningful patterns and trends from past data and predict future adherence, which can be used to design more personalized and effective cognitive training programs. These findings contribute to the growing body of research supporting AI-driven approaches for optimizing health care interventions in the aging population.

Our work demonstrates that SFDA techniques can effectively leverage information from external datasets and substantially improve the predictive accuracy of DL models, particularly when limited training data are available for developing personalized models for individual participants. More importantly, these methods offer the critical advantage of preserving data privacy, as they only require access to a model trained on a different clinical dataset, without requiring access to the raw data. Given strict privacy concerns in clinical applications and the ability of modern ML techniques to reidentify the deidentified data, training effective predictive models without direct access to the source data is a significant advancement. To the best of our knowledge, this study is the first to apply SFDA techniques to the domain of adherence prediction in cognitive training programs. The ability to maintain data privacy while still achieving competitive predictive accuracy has significant potential benefits in health care settings.

Furthermore, our study’s findings have important practical and theoretical implications for the design of future cognitive training interventions. By enabling accurate day-level adherence prediction from limited individual data, SFDA provides the foundation for just-in-time adaptive interventions that can detect early signs of disengagement. Early detection helps to deliver tailored support such as personalized reminders, motivational prompts, difficulty adjustments, and behavioral coaching. This predictive capability is critical for sustaining the prolonged engagement required for cognitive plasticity, as described in theoretical models of adult learning [12]. The results also indicate that adherence behaviors exhibit stable, transferable structural patterns, revealing a promising direction for using DA theory to model behavioral time-series and examine habit formation and individual differences in engagement. As SFDA enables cross-cohort knowledge transfer without accessing sensitive data, it supports the development of scalable, privacy-preserving personalized adherence support systems that can be deployed across sites and studies. From a practical standpoint, improving adherence prediction has direct implications for clinical trials, where consistent participant engagement is essential for accurately evaluating the effectiveness of cognitive training interventions. By improving the predictive accuracy of CNNs, SFDA-enhanced systems could make cognitive training trials more reliable, efficient, and informative.

### Limitations

Despite its advantages, applying SFDA in our study presents certain limitations. Deploying personalized models in real-world cognitive training applications raises several ethical and practical concerns, as users’ experiences with AI-health systems depend heavily on trust, privacy, and output interpretability [47]. Although SFDA ensures data privacy and personalized models enhance user engagement, it remains crucial to address issues related to user trust, transparency, and interpretability.

In addition to SFDA, other privacy-preserving techniques such as federated learning (FL) and homomorphic encryption exist. However, our focus on SFDA is motivated by its practicality in small-scale clinical studies. FL requires distributed infrastructure, multiple rounds of communication, and active collaboration among participants, increasing both computational burden and vulnerability to privacy attacks [48,49]. In contrast, SFDA involves sharing only a single source model without accessing source data or maintaining continuous client interactions. Hence, it is more suitable for our setting, in which trials are completed, and no live infrastructure exists for FL deployment.

Furthermore, there is a risk of domain mismatch when transferring personalized models to new cognitive games or different user populations. Models trained on limited individual-level data may overfit to short-term noise, reducing reliability. Moreover, tailoring models using only 30 days of data may introduce bias and restrict external generalizability, as early playing patterns may not fully capture long-term patterns. Finally, all 3 datasets used in our study were derived from the same cognitive training platform (Mind Frontiers) and involve participants with similar demographic and geographic characteristics, which may further limit generalizability. In future work, we will focus on evaluating SFDA across different cognitive training platforms, more diverse populations, and varied intervention types to assess the robustness and broader applicability of our approach.

### Conclusions

In conclusion, our research contributes to the growing body of research supporting AI-driven approaches to optimizing health care interventions for the aging population. Specifically, the findings support the use of DL and SFDA techniques for predicting adherence to cognitive training programs. With accurate adherence prediction models, AI-assisted personalized support systems can be developed to improve long-term engagement in cognitive training programs and maximize cognitive health benefits for older adults. We hope that our research will motivate further exploration of DA, particularly SFDA techniques in other contexts and populations, with the ultimate goal of improving the effectiveness of cognitive training interventions.

## Abbreviations

AI: artificial intelligence
CNN: convolutional neural network
DA: domain adaptation
DL: deep learning
FL: federated learning
LSC: local structure clustering
ML: machine learning
SFDA: source-free domain adaptation

## Data Availability

Data and analysis scripts will be made available upon request by contacting the corresponding author.
